# Evaluating Cross-Cutting Approaches to Chronic Disease Prevention and Management: Developing a Comprehensive Evaluation

**DOI:** 10.5888/pcd14.160499

**Published:** 2017-12-07

**Authors:** Marla Vaughan, Jan Jernigan, Seraphine Pitt Barnes, Pat Shea, Rachel Davis, Stephanie Rutledge

**Affiliations:** 1 Division for Heart Disease and Stroke Prevention, Centers for Disease Control and Prevention, Atlanta, Georgia

## Abstract

We provide an overview of the comprehensive evaluation of State Public Health Actions to Prevent and Control Diabetes, Heart Disease, Obesity and Associated Risk Factors and Promote School Health (State Public Health Actions). State Public Health Actions is a program funded by the Centers for Disease Control and Prevention to support the statewide implementation of cross-cutting approaches to promote health and prevent and control chronic diseases. The evaluation addresses the relevance, quality, and impact of the program by using 4 components: a national evaluation, performance measures, state evaluations, and evaluation technical assistance to states. Challenges of the evaluation included assessing the extent to which the program contributed to changes in the outcomes of interest and the variability in the states’ capacity to conduct evaluations and track performance measures. Given the investment in implementing collaborative approaches at both the state and national level, achieving meaningful findings from the evaluation is critical.

## Background

State Public Health Actions to Prevent and Control Diabetes, Heart Disease, Obesity and Associated Risk Factors and Promote School Health (State Public Health Actions) is a program funded by the Centers for Disease Control and Prevention (CDC) to support the statewide implementation of strategies that promote health and prevent and control multiple chronic diseases and their risk factors (1). In the program, CDC partners with state health departments to address the 4 domains of chronic disease prevention: 1) epidemiology and surveillance, 2) environmental approaches, 3) health care system interventions, and 4) community programs linked to clinical services (2). Four divisions in the National Center for Chronic Disease Prevention and Health Promotion (NCCDPHP) at CDC, the Division of Diabetes Translation (DDT), Division for Heart Disease and Stroke Prevention (DHDSP), Division of Nutrition, Physical Activity, and Obesity (DNPAO), and the School Health Branch (SHB) in the Division of Population Health, have collaborated to fund, implement, and evaluate State Public Health Actions.

Funding from the State Public Health Actions program has provided state health departments with an opportunity to address chronic diseases within their state at the individual level, such as by promoting health care interventions, and at the population level by developing policies and creating environments that promote health. This article is a companion to “Overview of State Public Health Actions to Prevent and Control Diabetes, Heart Disease, Obesity and Associated Risk Factors and Promote School Health,” which was published December 7, 2017, in *Preventing Chronic Disease* (3). Here we describe the approach taken to evaluate the collaborative, complex State Public Health Actions program to ensure its accountability by demonstrating health outcomes, assisting states and CDC in improving the implementation of programs, and expanding the body of practice-based evidence by identifying successful and replicable strategies.

## Evaluation Approach

Because State Public Health Actions is an innovative, cross-cutting program, it requires robust, multifaceted methods to evaluate it effectively. Although each of the 4 divisions conducted evaluations of their programs before State Public Health Actions, they took different approaches based on various factors, including the size and scale of the programs, the types of strategies being implemented (eg, policy, systems, and environmental changes, community-based and clinical interventions), and types of stakeholders engaged. Although evaluating large, federally funded public health programs is always challenging, the unique approach of State Public Health Actions compounded these challenges. Specifically, for State Public Health Actions there was a need to demonstrate to stakeholders its impact on disease-specific outcomes while implementing cross-cutting activities. Other challenges included coordinating across multiple chronic disease areas at the state and CDC level, accessing new partners and data sources, and the need to report performance measures that focused solely on outcomes.

**Figure 1:**
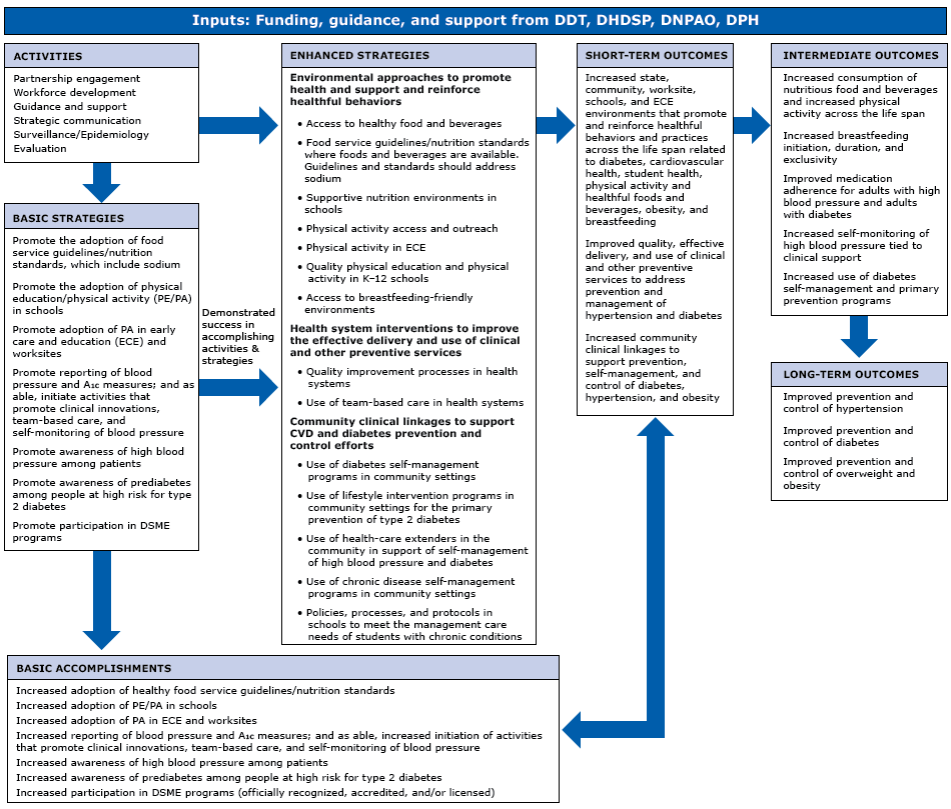
Program logic model for State Public Health Actions to Prevent and Control Diabetes, Heart Disease, Obesity and Associated Risk Factors and Promote School Health program. Abbreviations: A_1c_, glycated hemoglobin A_1c_; CVD, cardiovascular disease; DDT, Division of Diabetes Translation; DHDSP, Division for Heart Disease and Stroke Prevention; DNPAO, Division of Nutrition, Physical Activity, and Obesity; DPH, Division of Population Health, School Health Branch; DSME, diabetes self-management education; K–12, kindergarten through 12th grade.

These complex challenges required evaluators from each division to work together to design a comprehensive, multitiered approach to address the relevance, quality, and impact of State Public Health Actions. To begin, the evaluators followed standard practice by creating a logic model to highlight the inputs, activities, strategies, and outcomes of State Public Health Actions (Figure 1). The evaluators then designed the evaluation to assess and document the processes and outcomes of the program and to highlight how the implementation of the evidence-based strategies would lead to intended outcomes. The evaluation also examines the potential benefits and challenges of State Public Health Action’s approach of improving individual disease outcomes through the use of cross-cutting strategies.

The evaluation approach includes 4 primary components: conducting a national evaluation that assesses progress across all states; reporting by the states of performance measures to track the reach of individual strategies and disease-specific outcomes; conducting evaluations by the states to assess and improve programs at the state level and understand the facilitators of, and barriers to, program implementation; and providing evaluation technical assistance to enhance the capacity for evaluation at the local level and improve the reporting of data. CDC developed a structure to plan and implement the 4 components of the evaluation, which is to be carried out over a 5-year period. DHDSP was chosen to serve as the functional lead for evaluation in the administrative and management structure (3), while all 4 divisions identified a representative to act in a leadership role for evaluation-related decisions and the development of plans, processes, and guidance documents.

**Figure 2:**
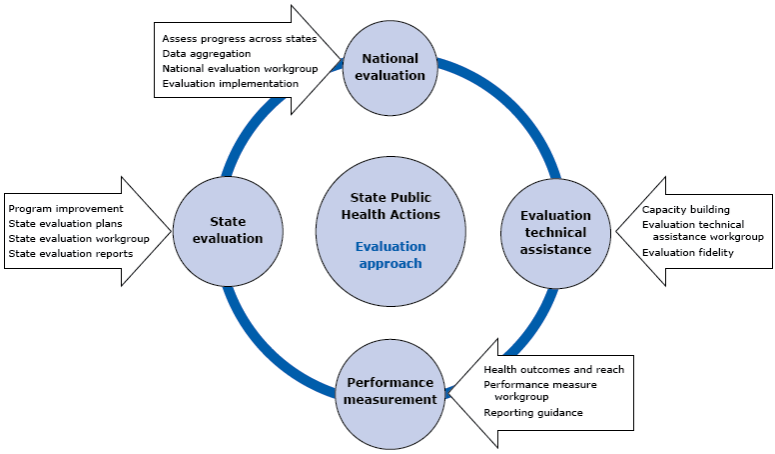
Components of state public health actions evaluation, State Public Health Actions to Prevent and Control Diabetes, Heart Disease, Obesity and Associated Risk Factors and Promote School Health (State Public Health Actions).

Four distinct evaluation workgroups were created to 1) oversee and implement the national evaluation; 2) collect, analyze, report, and provide guidance on performance measures; 3) provide guidance on planning and reporting the individual states’ evaluations; and 4) give technical assistance to build evaluation capacity among the states and ensure successful implementation of the 4 components of the evaluation (Figure 2). For each component, the workgroup members identified and addressed both common and unique challenges to developing and implementing that component.

## National Evaluation

The national evaluation is the key mechanism for understanding the progress, achievements, and challenges of the overall State Public Health Actions program. This component aims to not only assess the impact, effectiveness, and efficiencies of the program but also to determine the degree to which cross-cutting approaches affect outcomes for health promotion and chronic disease prevention.

### Development

The national evaluation workgroup used the CDC evaluation framework (4) to guide the evaluation’s design and methods and to provide context for the findings. The workgroup developed 4 overarching evaluation questions that will be assessed throughout the 5-year span of State Public Health Actions:

1. To what extent has the program been effective, as indicated by progress toward the intended accomplishments and outcomes?
2. To what extent, if any, have state programs gained efficiencies (eg, in infrastructure, management, financial performance) through the implementation of the approach of State Public Health Actions?
3. To what extent, if any, has CDC gained efficiencies by combining the efforts of 4 of its divisions within NCCDPHP?
4. What promising and innovative strategies that could be replicated by state programs have been found effective and efficient?

The 5-year national evaluation plan comprises an examination of the collaborations, efficiencies, activities, and accomplishments of all awardees; an in-depth analysis of the implementation and effectiveness of specific strategies; and an examination of the efficiency of CDC’s internal coordination and the effectiveness of technical assistance to awardees.

### Implementation

The national evaluation seeks to assess the implementation and outcomes of the program across all 50 states and the District of Columbia. Because the grantees are at different stages of implementation throughout the program period and because there are several potential focus areas and priorities, CDC evaluators develop an evaluation protocol for each year that incorporates programmatic priorities and subevaluation questions guided by the 4 overarching evaluation questions. Once a protocol is drafted, CDC obtains feedback from evaluators, states, CDC partners, and program staff members to ensure that the protocol is feasible and aligns with stakeholder needs. CDC relies on the primary and secondary collection of both quantitative and qualitative data. Specific data collection and analyses include conducting quantitative analyses of data on state performance measures; fielding surveys to assess the efficiency and collaboration of CDC and the states; implementing focus groups and key informant interviews; and reviewing training and technical assistance notes, state work plans, annual performance reports, and evaluation plans and reports written by the states. While the nature of evaluating a large program conducted by all the states limits the ability to attribute outcomes to the program because of the lack of comparison groups, the multiple sources of data collected allow for data triangulation to identify and assess trends and common themes in state progress. The evaluation of State Public Health Actions strives to show the reach of the program, methods of implementation, its synergy and coordination, and its impact in terms of contributing to improvements in disease-specific outcomes.

## Reporting of Performance Measures

Performance measures provide accountability by answering questions about what was achieved or, conversely, not achieved (5). For State Public Health Actions, performance measures provide key data for reporting outcomes to stakeholders and provide quantitative data that is incorporated into the national evaluation for assessing short-term and intermediate progress across each strategy being implemented by states. There were, however, several challenges to the implementation of reporting on performance measures. For example, previous programs funded by the 4 divisions within NCCDPHP did not require the reporting of outcome performance measures, many of the strategies that states are implementing as part of State Public Health Actions are new, many states were required to engage with new partners (eg, health care systems) and, as a group, states had varying capacity to collect and report measures and access data sources.

### Development

To develop the performance measures, leadership from the DHDSP, DDT, DNPAO, and SHB reviewed the purpose and intended outcome of each strategy in the logic model (Figure 1) to determine the areas and type of performance measures needed. Each division pulled most of its measures from previously developed and pilot-tested measures. For example, of the measures selected by the DHDSP, all but one were chosen from a prior multiyear project working with stakeholders to develop a menu of indicators for the control of high blood pressure. For State Public Health Actions, each performance measure aligns with a strategy or intervention that focuses on outcomes relevant to specific disease outcomes and the interests of stakeholders.

To ensure the reporting of high-quality data and to build capacity to collect and report performance measures at the state level, CDC developed guidance documents and provided webinars related to calculating the reach of the intervention and developing baseline and target values. CDC also developed operational definitions, also called profiles, for each of the performance measures; each 2-page profile defines and describes the purpose of the measure, unit of analysis, target population, and setting. It also describes how to calculate the measure, including the data sources to be used and the frequency of data collection, and provides additional resources and references (Appendix). CDC worked with the states to review and finalize the profiles. Once the profiles were disseminated, a tip sheet and considerations for reporting were provided to assist states with the reporting of data on performance measures.

### Implementation

In 2013, the states reported initial baselines and targets for strategy-specific performance measures. From 2015 until the end of the program (2018), states are required to report targets and annual progress for performance measures associated with their selected strategies and interventions. The states use a CDC-provided template that includes the measures required for a particular strategy, the prepopulated baseline (based on earlier reporting), targets for the current year and year 5, and actual data for the current year. Depending on the measure, the states report the data as a number, rate, percentage, or numerator and denominator. They also report the data source(s) and, as needed, provide notes that would give context to CDC for understanding the data during its analysis.

Each year, CDC’s performance measure workgroup assesses the quality of the state-reported data on these measures and the appropriateness of the analyses conducted (earlier, CDC had developed criteria for data quality and determined the type of analysis to be used for each performance measure). Data analysts at CDC use the criteria and inclusion and exclusion criteria for final cleaning and analysis of the data. Assessment of data quality also helps determine the performance measures for which the data are of sufficient quality to include them in the national evaluation and identifies measures that have widespread issues with quality. In addition, the process enables the provision of appropriate technical assistance to remedy those quality issues.

## Evaluations by the States

The evaluations performed by the individual states aim to provide data relevant to those states while also contributing to the national evaluation. States use data for purposes such as continuous program improvement and being responsive to local stakeholders. CDC uses these data for purposes such as synthesizing information on common strategies that states are using to identify and engage partners. This information provides a complete picture of progress on the performance measures, aids understanding of facilitators and barriers to implementation, and identifies potential best practices.

### Development

Acknowledging the difficulties of aggregating results from evaluations conducted by the states and other challenges in reporting their data, including varying capacities and a lack of standard data collection methods, CDC developed a set of core process evaluation questions and division-specific core outcome evaluation questions to facilitate the aggregation and cross-analysis of findings from the states for the national evaluation. States were also encouraged to develop additional evaluation questions and indicators to meet their own evaluation needs. The core process evaluation questions were related to their coordination with critical partners, their work across areas of chronic disease, their type of organizational structure, and their increased efficiencies obtained. The division-specific core outcome evaluation questions were related to progress made and both the barriers that they encountered and facilitators that aided selected strategies (Table). To reduce their burden and to focus the evaluation, states were required to evaluate only 1 strategy for each CDC division. States could also select whether they were in the adoption or implementation phase of the strategy. CDC designed a template that states could use to provide background information on the particular approach and strategies of the program, the selection of activities implemented, settings and target populations, key stakeholders and partners involved in the program planning and implementation, indicators developed to monitor progress toward achieving an answer to the process evaluation question, and the synergistic approach used to implement the program.

**Table:** Summary of Division-Specific Core Outcome Evaluation Questions for State Evaluations, the State Public Health Actions to Prevent and Control Diabetes, Heart Disease, Obesity and Associated Risk Factors and Promote School Health (State Public Health Actions) Program

Division Topic Area	Outcome Evaluation Question
Nutrition, Physical Activity, and Obesity	What are the key activities and/or resources considered critical to successful adoption/implementation of Healthier food retail venues or farmers’ markets in underserved areas? Food service guidelines/nutrition standards in priority settings? Interventions to create or enhance access to places for physical activity with an emphasis on walking through either state policies or pedestrian/transportation plans? Standards to increase physical activity in ECEs? Breastfeeding policies and practices? What are the major barriers and facilitators to adopting/implementing Healthier retail food venues or farmers’ markets in underserved areas? Food service guidelines/nutrition standards in priority settings? Interventions to create or enhance access to places for physical activity with an emphasis on walking through either state policies or pedestrian/transportation plans? Standards to increase physical activity in ECEs? Breastfeeding policies and practices?
School Health	What state activities have been effective in promoting Nutrition policy development and nutrition practice adoption among districts and schools? The development of CSPAPs among districts and schools? The implementation of policies, processes, and protocols in schools to meet the management and care needs of students with chronic conditions?
	What critical factors or activities influence the successful implementation of Nutrition policy and nutrition practice? CSPAP?
	What are the major facilitators and barriers in helping districts and schools Create supportive nutrition environments, such as partnerships (eg, MOUs) with the Department of Education? How were the barriers overcome? Develop CSPAPs, such as partnerships (eg, MOUs) with the Department of Education? How were the barriers overcome? Meet the management and care needs of students with chronic conditions? How were the barriers overcome?
	To what extent has implementation of nutrition policies and nutrition practices increased Access to healthier foods and beverages at school? The number of physical activity opportunities available to students during the school day? The management and care needs of students with chronic conditions?
Heart Disease and Stroke	What were the major facilitators and barriers in promoting implementation of Quality improvement processes, such as use of EHRs, in health care systems? How were the barriers overcome? Team-based care in health systems? How were the barriers overcome?
	How has the state promoted the use of health-care extenders in the community in support of self-management of high blood pressure? What were key facilitators and barriers?
	To what extent has the state effectively promoted implementation Of quality improvement processes, such as use of EHRs, in health care systems? Of team-based care in health systems?
	What factors at the state level are necessary to promote the use of health-care extenders in the community in support of self-management of high blood pressure?
	How has the relationship between the state health department, health care systems, and other QI/HIT partners in the state changed as a result of State Public Health Actions? Include the following aspects: The extent to which the state is able to obtain health systems data. Key facilitators and barriers to strengthening these partnerships.
	What policies/systems facilitated the support and promotion of Team-based care? The increased use of health-care extenders?
	To what extent have the QI processes influenced the quality, delivery, and use of clinical services for hypertension management among health systems?
	What policies/systems are needed for health care systems to effectively Implement team-based care? Increase the use of health-care extenders?
Diabetes	What were the major facilitators and barriers in implementing the 4 drivers during the start-up/implementation phase? How were the barriers overcome? For diabetes self-management education? For lifestyle intervention programs?
	What were the key activities critical to addressing disparities in the 4 drivers during the start-up/implementation phase? For diabetes self-management education? For lifestyle intervention programs?

Abbreviations: CSPAP, Comprehensive School Physical Activity Program; ECE, early care and education; EHR, electronic health record; HIT, health information technology; MOUs, memorandums of understanding; QI, quality improvement.

The division-specific outcome evaluation sections of the template included additional information on barriers and facilitators, an indicator table, a findings and results section for each disease-specific core outcome evaluation question, and a plan to disseminate the results of their evaluation to internal and external stakeholders.

### Implementation

States annually submit to CDC their plans for evaluation and the evaluation results obtained. Data are stored on an internal SharePoint (Microsoft Corp) site, where CDC evaluators review the data and determine how best to synthesize the data and pull out common themes. The data are triangulated with other data for the national evaluation and summarized. The information provided in the evaluation plans enables CDC evaluation technical assistance providers to understand the proposed methods and, thus, more effectively assist the states in conducting their own evaluations. Technical assistance providers can also provide information to the program team about common barriers and facilitators, which can be used to develop trainings and technical assistance to support and improve program implementation.

## Providing Evaluation Technical Assistance

The national evaluation, reporting of performance measures, and state evaluations all rely on data received by the states. Because the states have varying levels of capacity for evaluation, CDC must provide technical assistance to ensure effective reporting to the agency and to make sure that the state-level evaluations are providing information relevant to improving programs and meeting the needs of stakeholders. Because 4 divisions at CDC support the work of the state health departments, with each bringing its own body of expertise as it pertains to implementing disease-specific interventions, evaluators from all 4 of these divisions have worked collaboratively as part of regional teams that support the states to evaluate various strategies they are implementing.

### Development

CDC’s technical assistance plan for the 5-year evaluation consists of evaluation capacity assessments, annual reviews of documents, the development of evaluation tools and resources, and other forms of technical assistance to the states. Evaluation capacity assessments were performed in the first year to understand the capacity of each state to conduct evaluations and to identify needs for technical assistance and types of trainings and resources that were needed for states to meet evaluation requirements. Ongoing assessments are also conducted to identify facilitators of and barriers to developing evaluation plans and tools, identifying appropriate indicators and data sources, and conducting data analysis for annual evaluation reporting. Evaluators at CDC maintain regular communication with evaluators at the state level and assist them with developing their evaluation plans, collecting and reporting performance measures, and reporting the results of their evaluations. CDC evaluators also assist both the states and project officers at CDC through the annual review of work plans, yearly performance reports, and evaluation reports to ensure that states are aligning activities with performance measures and accurately reporting data.

### Implementation

Evaluation resources made available to the states by CDC include training opportunities such as cross-state peer-learning communities, evaluation guidance documents, sample data collection tools, and evaluation plan and report templates. The peer-learning communities meet monthly for presentations and facilitated discussion. In addition, there is a listserve on which community members can pose questions to other members about their experiences implementing their evaluations and can share information and documents. Additional evaluation guidance documents and tools developed by CDC include templates and helpful hints documents to support the states’ work throughout various phases of the program.

Consistent and coordinated communication with states and among CDC staff is important to reaching the goal of providing effective technical assistance. To standardize technical assistance, evaluators developed a guide designed to support consistent monitoring and documentation of evaluation technical assistance needs for a state during evaluation plan implementation and performance measure reporting. In collaboration with project officers, evaluators at CDC communicate with states at least monthly through regular calls with the regional team and ad hoc, evaluation-specific follow-up calls and email communication. Internally, CDC uses a performance-monitoring database to document progress on performance and evaluation activities and to track communication and follow-up activities between the states and CDC’s evaluation staff and project officers.

## Dissemination and Use of Evaluation Findings

CDC regularly disseminates findings related to the evaluation of State Public Health Actions to various stakeholders, including internal and external partners as well as the general public, through reports, executive summaries or briefs, presentations, and journal publications. Reports internal to CDC are used to understand how states are implementing programs and how well CDC is providing technical assistance to states and coordinating across divisions. Briefing documents, such as the State Public Health Actions Year 3 Performance Measures Snapshots (6), and the DNPAO state snapshots website (7), which report on highlights at the state level, are used to provide information on the program’s priorities and offer succinct outcomes that are relevant to stakeholders. Findings are also prepared for national partners and Congress to demonstrate accountability and program impact.

Presentations of findings are delivered internally to the CDC staff and externally to state health departments’ staff and other public health practitioners. For example, presentations were made at a meeting of grantees in Atlanta, Georgia, and to various diverse audiences at national conferences, such as those that were held by the American Public Health Association and the American Evaluation Association (8). Evaluation methods and findings obtained are also being shared through journal articles written by the CDC staff and state representatives (3, 9–11). In addition, CDC provides assistance to states in writing journal articles and finding strategies for dissemination.

## Conclusion

The approach to the evaluation of State Public Health Actions is intended to demonstrate the impact of the overall program while capturing unique cross-cutting aspects of the program and the disease-specific outcomes. Lessons learned and key findings from the national evaluation, performance measures data, and evaluations conducted by the states will be summarized throughout the 5 years of the program to assist with ongoing program improvement, report progress to stakeholders, identify successful strategies, and inform future decisions on funding. While the comprehensive evaluation strives to evaluate efficiency, effectiveness, and impact at the state and national levels, it faces numerous challenges.

Evaluations of large public health programs are difficult to conduct, with one of the big challenges being the inability to attribute successes or shortfalls wholly to the program, because there are often confounding factors, a lack of comparison groups, long time frames, or multiple interventions going on at once. The development and use of performance measures to assess outcomes for federal programs is also challenging because of issues such as the complexity of public health problems, which may have multiple determinants or outcomes that may take several years to achieve; the decentralized implementation of public health programs; and measurement issues related to a lack of reliable, timely, and consistent data sources (5). Also, to successfully aggregate standardized measures, it would be ideal, but not realistic, for the states to have similar capacities to access, collect, analyze, and report data. Finally, federal agencies are challenged by the limited resources available to provide state health departments with consistent and intensive technical assistance with evaluation to help them with collecting and reporting performance measures and evaluating their programs.

These common challenges are clearly applicable to State Public Health Actions, with the added complexity of working across multiple topic areas and attempting to evaluate cross-cutting strategies when most state health departments and CDC operate within distinct disease or topic areas. Each topic area has discrete funding streams and must demonstrate effectiveness in achieving outcomes for each of these areas. The State Public Health Actions program also expands funding to more states than were previously funded by each division, and oversight and management requires complex coordination. To accurately describe the implementation and outcomes of State Public Health Actions, assessing collaboration and coordination across topic areas at the state level and at CDC is an important part of the evaluation. Surveys, focus groups, key informant interviews, and results obtained from evaluations conducted by the states using a standard template are employed to highlight this unique aspect of State Public Health Actions. CDC evaluators provide proactive and intensive technical assistance to address challenges, but the complex, cross-topic, structure of technical assistance can be time-consuming.

Although there are challenges and limitations with the evaluation of State Public Health Actions, given CDC’s substantial investment in testing collaborative approaches and working across domains, striving to achieve meaningful findings from the evaluation is critical. Subsequent articles will highlight results achieved by the program and promising practices that can be implemented broadly.

## Appendix. Sample Performance Measure Profile for State Public Health Actions to Prevent and Control Diabetes, Heart Disease, Obesity and Associated Risk Factors and Promote School Health (State Public Health Actions) Program

This file is available for download as a Microsoft Word document.
