# Supplementary material for: Evaluating Cross-Cutting Approaches to Chronic Disease Prevention and Management: Developing a Comprehensive Evaluation
Source: Prev Chronic Dis. 2017 Dec 7;14:E131. doi: 10.5888/pcd14.160499 (PMC5725148; doi:10.5888/pcd14.160499)
Supplement: Supplementary file 1 [file 16_0499-appendix.doc]

Appendix. Sample Performance Measure Profile for State Public Health Actions to Prevent and Control Diabetes, Heart Disease, Obesity and Associated Risk Factors and Promote School Health (State Public Health Actions) Program

| **CDC Operationalized 1305 Performance Measure** | |
| --- | --- |
| **Date:** | |
| **Performance Measure:** Proportion of health care systems with electronic health records (EHRs) appropriate for treating patients with high blood pressure  **Note this performance measure has been separated into two (one for high blood pressure and one for diabetes):* Proportion of health care systems with electronic health records (EHRs) appropriate for treating patients with diabetes. The diabetes profile is currently being developed. | |
| **Strategy: 1:** Increase implementation of quality improvement process in health systems | |
| **Intervention *(Enhanced only)*:**   - Increase EHR adoption and the use of health information technology (HIT) to improve performance - Increase the institutionalization and monitoring of aggregated/standardized quality measures at the provider and systems levels | |
| **□ Basic** | |
| **☒ Enhanced** | |
| - Domain 2: Environmental Approaches that Promote Health   ☒ Domain 3: Health Systems Interventions   - Domain 4: Community Clinical Linkages | |
| ☒ Short Term ☐ Intermediate ☐ Long Term | |
| **AREAS** | **EXPLANATION** |
| **Purpose of Performance Measure** | - The purpose of this performance measure is to look at health care systems that have EHRs appropriate for treating patients with high blood pressure. Effective use of information and medical technology is one of the strategies the Institute of Medicine (2001) recommended to improve the quality of care in the U.S. There is both direct and indirect evidence that shows electronic health records systems improve high blood pressure (HBP) management by enhancing health information exchange between patients, providers, and health systems (Kinn et.al, 2002; Rossi & Every, 1997). |
| **Results Statements** | - In the US, CDC funded grantees worked to increase the number and proportion of health care systems that are equipped with and use EHRs that are appropriate for treating patients with high blood pressure from (# and %) to (# and %). This represents an increase of % over the funding period. - In the US, CDC funded grantees worked to increase the number and proportion of systems that use computer-based clinical decision support systems to treat patients with high blood pressure from (# and %) to (# and %). This represents an increase of % over the funding period. |
| **Definition of Terms** *(Key concepts defined)* | - Appropriate Electronic Health Records = Certified EHR by the Office of the National Coordinator for Health Information Technology (ONC). EHRs can support implementation of evidence-based treatment guidelines through clinical decision supports (CDS), alerts, patient reminders, and patient registry functionality. Feedback reports/dashboards allow providers to manage individuals and entire patient panels through targeted interventions for those in need of more intensive care. EHRs with information-exchange functionality can improve coordination of care within the health care system and with community support and prevention services. - Meaningful Use is an effort led by the Centers for Medicare & Medicaid Services (CMS) and the ONC to promote the effective use of EHRs. Some meaningful use objectives that could improve chronic disease outcomes include: incorporating clinical lab test results as structured data, generating lists of patients by specific conditions, medication reconciliation, and sending reminders to patients in their preferred method about preventive or follow-up care. Health care systems = Health care delivery organizations including health maintenance organizations (HMOs), Federally Qualified Health Centers (FQHCs), Rural Health Centers (RHCs), and other clinical groups operating within the state. In instances where the state government or local government is responsible for providing clinical care, these settings could be included. - High Blood Pressure (HBP) = Systolic blood pressure (SBP) of 140 mm Hg or higher or diastolic blood pressure (DBP) of 90 mm Hg or higher (CDC, 2013).   Patients = Adults aged ≥18 years old |
| **Unit of Analysis** | Health care systems equipped with EHRs appropriate for treating high blood pressure |
| **Intended/Targeted Population** | Defined health care systems |
| **Numerator** | Number of defined health care systems that use EHRs appropriate for treating patients with high blood pressure |
| **Denominator** | Total number of health care systems in the state (or at the highest level possible) |
| **Rate/Count/Percentage** | N.A. |
| **Disparities Focus** | **Approach related/specific:** N.A. |
|  | **Stratification:** N.A. |
| **Data source(s)** | **The following data sources can be used to collect data for this measure:**   - Health Resources and Services Administration (HRSA) Uniform Data System (UDS) <http://bphc.hrsa.gov/healthcenterdatastatistics/reporting/index.html> - The Office of the National Coordinator for Health Information Technology (ONC) Product List <http://oncchpl.force.com/ehrcert?q=chpl> - ONC dashboard <http://dashboard.healthit.gov/HITAdoption/?view=0> |
|  | **Not Appropriate:**   - An individual local practice |
| **Frequency of Data Collection** | Annually |
| **References** | - Centers for Disease Control and Prevention. *Self-Measured Blood Pressure Monitoring: Action Steps for Public Health Practitioners.* Atlanta, GA: Centers for Disease Control and Prevention, US Dept of Health and Human Services; 2013. - Kinn JW, Marek JC, O’Toole MF, Rowley SM, & Bufalino VJ. Effectiveness of the electronic medical record in improving the management of hypertension. *J Clin Hypertens* 2002;4(6):415-9 - Rossi RA & Every NR. A computerized intervention to decrease the use of calcium channel blockers in hypertension. *J Gen Intern Med* 1997;12*:*672-678 - FOA website: [http://www.cdc.gov/chronicdisease/about/statepubhealthactions- prevCD/faq/index.htm](http://www.cdc.gov/chronicdisease/about/statepubhealthactions-prevCD/faq/index.htm) |
| **Questions/Issues for CDC Grantee Collaboration** | N.A. |
| **Additional Information/Guidance** | CMS offers an incentive payment to eligible professionals or hospitals that demonstrate they have engaged in efforts to adopt, implement, or upgrade certified EHR technology. Meaningful Use criteria are designed to standardize what functionality EHR systems must have. Starting in 2014, eligible professionals will be required to meet 9 out of 64 possible clinical quality measures, and blood pressure control is highlighted as recommended core measure.  Meaningful Use Stage 2 requires that (1) providers use 5 clinical decision–support interventions related to 4 or more clinical quality measures (e.g., an ABCS measure), (2) at least 10% of active patients are sent a reminder for a clinical appointment or service, (3) EHRs are used to identify educational resources for patient-specific needs and these resources are provided to more than 10% of patients, and (4) at least 5% of patients receive secure electronic messaging (e.g., secure e-mail, patient portal). |
